# Red and near-infrared light-activated photoelectrochemical nanobiosensors for biomedical target detection

**DOI:** 10.1007/s00604-024-06592-x

**Published:** 2024-08-14

**Authors:** Yeison Monsalve, Andrés F. Cruz-Pacheco, Jahir Orozco

**Affiliations:** Max Planck Tandem Group in Nanobioengineering, Institute of Chemistry, Faculty of Natural and Exact Sciences, University of Antioquia, Complejo Ruta N, Calle 67 No. 52-20, 050010 Medellín, Colombia

**Keywords:** Photoelectrochemical nanobiosensor, Photoactive nanomaterial, Red light, Near-infrared (NIR), Analytical performance

## Abstract

Photoelectrochemical (PEC) nanobiosensors integrate molecular (bio)recognition elements with semiconductor/plasmonic photoactive nanomaterials to produce measurable signals after light-induced reactions. Recent advancements in PEC nanobiosensors, using light-matter interactions, have significantly improved sensitivity, specificity, and signal-to-noise ratio in detecting (bio)analytes. Tunable nanomaterials activated by a wide spectral radiation window coupled to electrochemical transduction platforms have further improved detection by stabilizing and amplifying electrical signals. This work reviews PEC biosensors based on nanomaterials like metal oxides, carbon nitrides, quantum dots, and transition metal chalcogenides (TMCs), showing their superior optoelectronic properties and analytical performance for the detection of clinically relevant biomarkers. Furthermore, it highlights the innovative role of red light and NIR-activated PEC nanobiosensors in enhancing charge transfer processes, protecting them from biomolecule photodamage in vitro and in vivo applications. Overall, advances in PEC detection systems have the potential to revolutionize rapid and accurate measurements in clinical diagnostic applications. Their integration into miniaturized devices also supports the development of portable, easy-to-use diagnostic tools, facilitating point-of-care (POC) testing solutions and real-time monitoring.

## Introduction

PEC nanobiosensors use nanoscale components and light-matter interaction to provide specific quantitative or semiquantitative analytical information about a (bio)analyte. They convert biological signals into electrical signals under the influence of light. PEC nanobiosensors consist of nanostructured components linked to a molecular recognition element or bioreceptor that specifically binds to the analyte and a transducer that converts this interaction into a measurable electrical signal [1, 2]. Rather than referring to the nanometric size of the entire device, the term nanobiosensor in this review refers to a system with at least one nanostructure within its components [3], whose enhanced properties from the nanoscale dimension give place to new, improved features and functionalities when assembled into biosensing devices [4]. PEC nanobiosensors utilize the interaction of light with photoactive materials to follow electrochemical reactions, benefiting from enhanced charge separation and signal amplification [5]. They typically integrate molecular recognition elements and/or (bio)receptors (e.g., enzymes, antibodies, nanobodies, peptides, cellular receptors, nucleic acids, glycans, aptamers, among others) with photoactive nanomaterials (e.g., semiconductor and plasmonic materials) [6–9]. Characterized by their high sensitivity and specificity, PEC nanobiosensors offer significant advantages such as signal amplification, minimal background noise, and reduced photodamage. These sensors feature tunable optical properties, photostability, durability, and amenability for surface functionalization. By leveraging PEC approaches, these biosensors provide rapid response times, versatility, and multi-functionality [10]. The current or voltage response under irradiation with light of different wavelengths in PEC biosensors changes when a recognition event occurs on the transducer surface or electrode [11]. It allows for highly specific and sensitive detection of various analytes, making PEC biosensors a promising tool for diverse applications in medical diagnostics [12]. Additionally, PEC nanobiosensors can be manufactured rapidly and cost-effectively for single-use devices, enabling efficient measurement collection using disposable electrodes, simplifying sensor handling, reducing contamination risks, and eliminating laborious cleaning or maintenance steps [13]. This combination of high sensitivity, miniaturization, and disposable amenability makes PEC biosensors well-suited for rapid, cost-effective, and user-friendly bioanalytical applications [14].

Incorporating high-surface-area photostimulable nanomaterials onto transducer platforms has further enhanced the performance of PEC sensing devices [15]. These nanomaterials can improve energy transfer processes, amplifying transduction signals to achieve highly sensitive, stable, and reproducible devices [16]. In particular, plasmonic nanoparticles, such as noble metals like gold (Au) and silver (Ag), exhibit collective oscillations of free electrons on their surface. This phenomenon leads to the absorption, scattering, and amplification of electromagnetic signals in the visible and NIR regions of the spectrum [17]. Utilizing this spectral radiation range to stimulate plasmonic nanoparticles in PEC biosensing is advantageous, as it minimizes potential photodamage to biomolecules compared to ultraviolet (UV) radiation [18, 19]. By harnessing these advancements, plasmon nanoparticle-based PEC biosensors offer improved stability and analytical performance without compromising biointerface integrity, thereby facilitating sensitive analyte detection [20]. In PEC detection, light is crucial in exciting the photoactive species, generating an electrical signal for transduction, and facilitating the detection process [21]. Separating the excitation source from the detection system endows this technique with potentially higher sensitivity. This heightened sensitivity is specifically due to the ability to automate the system’s excitation source, allowing it to be turned on and off in a specific time window. This automation enables a precise response to the detection of the analyte of interest, effectively eliminating background noise from secondary reactions that do not correspond to the PEC detection event of the system [22, 23]. Moreover, the ease of miniaturizing PEC biosensing systems renders them more effective than conventional optical and electrochemical methods [24, 25]. This efficacy is due to the favorable photogenerated charge transfer reactions at the modified electrode surface [26]. When the analyte is present in the sample, the resultant specific recognition events can directly or indirectly induce alterations in the PEC signal, used to monitor the analyte levels [27, 28].

The selection of the photoactive material stands out as one of the most critical steps in determining the analytical performance of PEC devices. This choice is vital for enhancing charge conversion at the photoactive surfaces [1]. In recent years, semiconductor nanomaterials have emerged as the most utilized photoactive materials for PEC biosensing applications [29]. Various factors influence the performance of PEC devices, including changes in the photon conversion properties of typical semiconductor materials employed in transducer platforms. These factors encompass temperature fluctuations, external light exposure, electric and magnetic fields, and alterations in their electronic states of valence and conduction bands [30–33]. Such changes result in a sensitive response and impart unique properties in photoelectricity, photoluminescence, electroluminescence, electrochemiluminescence, and thermoelectric phenomena [34–38]. Semiconductor nanostructures exhibit a robust absorption capacity and an inherent electronic band structure [39]. Innovations in semiconductor morphology, structure, or elemental composition can bolster charge transport, facilitating high photoelectric conversion efficiency [40, 41].

Even though plenty of reviews have already been reported in the literature [42–45], there is still a knowledge gap intended to fill in this topic. This work reviews the crucial role of PEC nanobiosensors in detecting a wide spectral range of bio-analytes, discussing their impact on analytical performance. It compares PEC detection approaches stimulated by the spectrum’s red light and NIR regions and thoroughly outlines the technical characteristics of these PEC assays, including their physicochemical properties, signal sources, sensing formats, and signaling strategies. Additionally, it explores various photoactive nanomaterials currently employed in PEC applications, examining their compositional and structural properties to enhance biosensing methodologies for various bio-analyte detection scenarios. Finally, it showcases the potential of red light and NIR region sources to improve PEC performance and finalizes with concluding remarks and perspectives to better exploit transduction mode PEC-based devices.

## Technical characteristics of photoelectrochemical biosensors

PEC explores the interaction between light and photoactive materials, resulting in the interconversion of photoelectric and chemical energy [46]. The physical interaction between the photoactive material and the electrode promotes the charge transfer generated by the photons absorbed from the material, producing electrons and holes. Sacrificial reagents or redox mediators in solution transfer electrons to the photogenerated holes to restrict charge recombination in the material. The charge transfer on the electrode is reflected in an increase in current or potential resulting from excitation with light [47]. PEC biosensors integrate photoactive materials and molecular biorecognition elements (bioreceptors) coupled to the electrode interface to detect various (bio)analytes [48]. The change of the PEC signal when the electrode is exposed to defined spectral ranges of light irradiation evidences the biorecognition event between the bioreceptor and the target (bio)analyte.

**Fig. 1:**
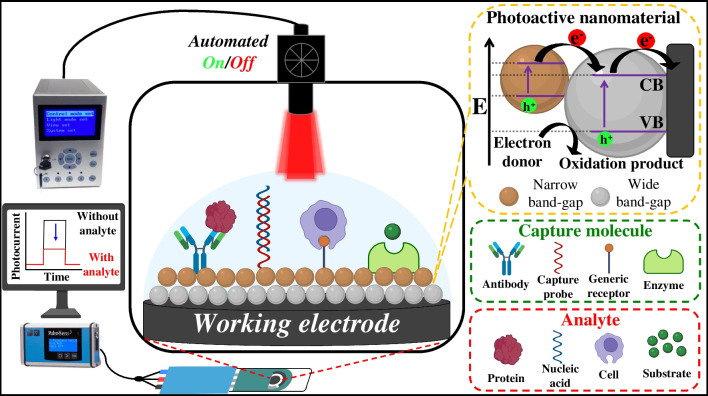
Schematic representation of PEC assays utilizing miniaturized electrochemical cells, external excitation sources, and specific interactions in immunosensing, genosensing, enzymatic, and cytosensing assays. Charge generation and transduction occur at the electrode surface through photoactive nanomaterials promoted by the alignment of conduction (CB) and valence bands (VB) in materials with varying band-gaps

Conventional photodetection systems encompass four key components, as illustrated in Fig. 1. First is the excitation source (light source), followed by the signal transduction platform, which consists of the electrode, photoactive material, and molecular recognition elements. The third component is related to the redox mediator dissolved in an electrolytic medium. Finally, the PEC signal-reading system [49]. Multiple interconnected physical and chemical processes are essential to generate the signal. Initially, photons are absorbed, initiating a charge separation process in the material. Subsequently, charges migrate and recombine at the interface between the photoactive material on the working electrode and the redox mediator [50].

Efficiently converting photons into electric charge is a crucial factor in PEC processes [51, 52]. PEC devices integrate light as an excitation source to generate an increased electrical signal, improving sensitivity compared to conventional electrochemical processes [53, 54].

### Photoactive nanomaterials

A photoactive nanomaterial can generate chemical or physical changes when interacting with electromagnetic radiation, usually in detection systems in the ultraviolet–visible (UV–vis) and NIR regions [55, 56]. The functionality of a photoactive material involves the absorption of light energy, the generation of electron–hole pairs, and a specific response that depends on its structural properties and the surrounding medium [57]. Integrating nanostructured materials into PEC biosensors offers advantages, including increased surface area, improved PEC features, bioconjugation, enhanced analytical properties, and the potential for miniaturization and amenability for portable sensing devices [58]. The light-sensitive nanostructured material interacts closely with the electrode and facilitates the transduction of the biochemical interaction into a quantifiable electrochemical signal [59]. The choice of a photoactive nanomaterial depends on the requirements of the PEC sensing application, encompassing the target analyte, detection sensitivity, and operating conditions [60]. Consequently, research on new photoactive materials would reinforce the versatility and functionality of PEC detection in bioanalysis applications [61, 62].

### Physicochemical considerations

Specific physicochemical parameters play a pivotal role in comprehending the performance of photoactive nanomaterials [63]. To effectively absorb electromagnetic radiation and generate charge carriers leading to PEC detection, these materials must initially possess optical properties, including energy absorption and emission, as well as high quantum yield and extinction coefficient [64]. The morphology, atomic configurations, and nanostructure’s exposed surface area are related to the efficient transfer of charge carriers during reactions in electrolytic media [65].

Achieving proficient charge transfer and efficient electron flow within a PEC system hinges on the alignment of energy levels between photoactive nanomaterials and other components [66]. This alignment is crucial for effective charge injection, transport, and collection at the electrode. Equally important is selecting the appropriate excitation wavelength range and the energy level at which the nanomaterial is stimulated [67]. The range of wavelengths that photoactive nanomaterials absorb depends on their band-gap [68]. The feasibility of designing and manipulating this band-gap in PEC applications is demonstrated through doping, alloying, or quantum confinement effects. These methods allow absorption spectra adjustment and maximization of nanomaterial photoactivity [69].

### Sources of signals and excitation

The photocurrent signals produced by PEC biosensors involve various kinetic and thermodynamic steps [70]. The performance of PEC biosensors is influenced by light excitation, photogenerated carrier transfer, and redox mechanisms [71, 72]. According to energy band theory, electrons are propelled from the valence band (VB) to the conduction band (CB) when photons with energy equal to or exceeding the band-gap energy (E_g_) of the photoactive nanomaterial irradiate them [73]. These photogenerated carriers are then transported to the electrode or electrolyte, but their effective utilization requires their migration to the surface from within the material [74]. Upon the creation of the electron–hole pair, a fraction of carriers promptly recombines, while others do so during their journey to the surface (as illustrated in Fig. 1). Carrier migration, a relatively slow process, introduces varying recombination pathways. Upon reaching the surface, carriers might engage in redox reactions with electroactive species in the electrolyte [75]. Nonetheless, many carriers recombine on the surface before completing these processes due to the time-consuming nature of electroactive species adsorption and medium-related redox reactions. The migration of carriers and the rates of reactions in photoactive materials are influenced by the VB/CB levels and the redox potential of electroactive species [76]. From a thermodynamic standpoint, oxidation/reduction reactions occur when the oxidizing species potential is more positive than the CB level and the reducing species potential is more negative than the VB level [77].

### (Bio)sensing formats and signaling strategies

The construction of PEC biosensing assays has proven challenging in developing novel photoactive nanomaterials and searching for more sensitive, precise, and accurate signals [78]. Highly specific and selective detection formats have achieved tests with minimal background noise compared to conventional methodologies [79]. The specificity and selectivity of the bioreceptor and the stable coupling with photoactive nanomaterials are paramount factors for direct detection of the molecular target [80, 81]. Consequently, PEC analysis’s versatility and practical potential have found widespread applications in many scientific domains, particularly in identifying various (bio)analytes of biochemical and clinical interest [82]. These applications encompass nucleic acid analysis [44], immunoassays [83, 84], cell detection [85–87], enzyme and protein bio-detection [88–90], and monitoring of small (bio)molecules [91, 92].

Nevertheless, PEC detection presents a massive challenge in sensitively detecting various (bio)analytes, particularly those with exceedingly low levels, such as biomolecules, during the early stages of diseases. This reality places heightened demands on PEC sensors’ sensitivity and detection range [11]. Therefore, numerous signal amplification strategies have been introduced to enhance the practical utility of the devices. High analytical performance, self-powered functionality, and miniaturization significantly impact the overall effectiveness of PEC detection systems [93]. Likewise, detecting multiple analytes and analyzing big data are other progressive needs that require customization of detection systems [94]. Consequently, the research on PEC biosensors has a noteworthy influence on endeavors to innovate and elevate the functionality of these devices [95, 96].

## Classification of photoactive nanomaterials

Over the last decade, nanomaterials capable of interacting with electromagnetic radiation in the UV–visible and NIR ranges have been successfully coupled into PEC applications, highlighting photocurrent and photopotential signals [97, 98]. Table 1 overviews the critical characteristics of various materials used in PEC detection processes. PEC systems generally require a redox probe to reveal the generated photocurrent and complete the charge transport cycles. Most semiconductor systems used in PEC systems have well-defined band-gap values to determine the optimal excitation energy ranges. While band theory elucidates the general PEC principle, most PEC assays involve different optical and electrical phenomena depending on the nanomaterial and photoactive nanomaterials arranged on the transduction surface. In this context, molecular biorecognition events involve different PEC detection mechanisms. This review classifies PEC systems according to the photoactive nanomaterial type, including metals and metal oxides, carbon nitrides, quantum dots, semiconductors, and transition metal chalcogenides (TMCs).

**Table 1:** Classification of photoactive nanomaterials in UV- and visible-light-activated PEC biosensors

PEC materials	Platform structure	Electrolyte/redox probe	*λ*_exc_ (nm)	Band-gap (eV)	Detected biomarker	Linear range	LOD	Ref
*Metals and metal oxides*	Au@ZnO/FTO nanorods	GSH/GSSG–PBS	1 sun	**-**	GSH	20–1000 µM	3.29 µM	[99]
	ZnONRs/TNs/TiO	(NH_4_)_2_SO_4_	≥ 420	2.89	AChE	0.05–1000 µM	0.023 µM	[100]
	dTiO_2−*x*_@Au	Exo lll/PBS	585	2.52	DNA	1 pM–10 nM	0.6 pM	[101]
	Au/GR-CdS	Na_2_SO_4_	Xe Lamp	**-**	Diclofenac	1–150 nM	0.78 nM	[102]
	PdO/APFO-3: PCMB	NaHCO_3_-PBS	1 sun	**-**	Oxygen	0.5–20 mg/L	0.034 mh/L	[103]
*Carbon nitrides*	g-C_3_N_4_/Co_3_O_4_	Na_2_HPO_4_/NaH_2_PO_4_	Xe lamp	2.62/2.13	Oxytetracycline	0.01 – 500 nM	3.5 pM	[104]
	g-C_3_N_4_/AuNPs/CoO	Na_2_SO_4_/PBS	> 420	2.75/2.85	Microcystin-LR	0.1 pM – 10 nM	0.01 pM	[105]
	g-C_3_N_4_/BiVO_4_	PBS	> 420	2.70/2.40	Microcystin-LR	5 pg/L – 10 µg/L	41.9 fg/L	[106]
	g-CNS3	AA/PBS	Xe lamp	2.59	ALV-J	10^2.14^–10^3.35^ TCID_50_/mL	10^2.08^ TCID_50_/mL	[107]
	g-C_3_N_4_/TiO_2_	AAP/PBS	> 460	2.69/3.21	Protein kinase A	0.05 – 100 U/mL	0.048 U/mL	[108]
*Quantum dots*	g-C_3_N_4_/CdS QDs	AA/PBS	Xe lamp	2.42	Prostatic antigen	0.01 – 50 ng/mL	4 pg/mL	[109]
	g-C_3_N_4_/CdS QDs	AA/NaCl-KCl	Xe lamp	2.42	MicroRNA-21	0.1 fM – 1 nM	0.05 fM	[110]
	rGO/CdS QDs	H_2_O_2_/PBS	> 450	**-**	2,3′,5,5′ Tetrachlorobiphenyl	10–1000 ng/mL	1 ng/mL	[111]
	h-BN/CdS QDs	AA/PBS	Xe lamp	-	MicroRNA-141	0.001–100 nM	0.73 fM	[112]
	WS_2_/β-CD@CdS nanorod	AA/PBS	1 sun	1.46/2.36	MicroRNA-21	0.1 fM – 10 pM	25.1 aM	[113]
*Transition metal chalcogenides*	Single-layer nanoMoS_2_	PBS	White LED	-	Dopamine	10 pM – 10 µM	2.3 pM	[114]
	SnS_2_@Ti_3_C_2_	Tris–HCl	Xe lamp	1.86	5cadCTP	0.001 – 200 nM	260 fM	[115]
	MoS_2_/NGQDs	PBS	Xe lamp	**-**	Acetamiprid	0.05 pM – 1 nM	16.7 fM	[116]
	WS_2_/MoS_2_/β-TiO_2_	AA/PBS	> 420	1.37/1.57/2.38	5-Formylcytosine	0.01–200 nM	2.7 pM	[117]
	CdS/SnS_2_/CNTs/GCE	PBS	Xe lamp	2.12/1.92	Hydroquinone	0.2–100 μM	0.1 μM	[118]

*5cadCTP*, 5-carboxy-2′-deoxycytidine-5′-triphosphate; *AA*, ascorbic acid; *AChE*, acetylcholinesterase; *Ag*_*2*_*S*, silver sulfide; *AgI*, silver iodide; *ALV-J*, J avian leukosis virus; *APFO-3*, ammonium pentadecafluorooctanoate; *Au@ZnO/FTO*, heteroconjuction of gold nanoparticles, zinc oxide, and fluorine-doped tin oxide; *Au/GR-CdS*, heteroconjuction of gold nanoparticles, reduced graphene, and cadmium sulfide; *AuNPs*, gold nanoparticles; *BiOBr*, bismuth oxybromide; *BiVO*_*4*_, bismuth vanadate; *BN*, boron nitride; *CdS QDs*, cadmium sulfide quantum dots; *CdS/SnS*_*2*_*/CNTs/GCE*, heteroconjuction of cadmium sulfide, tin disulfide, carbon nanotubes, and glassy carbon electrode; *CN*, carbon nitride; *CoO*, cobalt(II) oxide; *DNA*, deoxyribonucleic acid; *dTiO*_*2-x*_*@Au*, titanium dioxide and gold nanoparticles composite; *Exo III*, exonuclease III enzyme; *[Fe(CN)*_*6*_*]*^*3−/4−*^, hexacyanoferrate; *GSH*, glutathione; *GSSG*, oxidized glutathione; *g-C*_*3*_*N*_*4*_, graphitic carbon nitride; *g-C*_*3*_*N*_*4*_*/Co*_*3*_*O*_*4*_, heteroconjuction of graphitic carbon nitride and cobalt(II) oxide; *g-CNS3*, three-step thermal polycondensation of 2D g-C_3_N_4_ nanolayers; *ITO*, indium tin oxide; *KCl*, potassium chloride; *LOD*, limit of detection; *MgCl*_*2*_, magnesium chloride; *MCF-7*, breast cancer cell line; *MoS*_*2*_, molybdenum disulfide; *Na*_*2*_*SO*_*4*_, sodium sulfate; *NaHCO*_*3*_, sodium hydrogen carbonate; *(NH*_*4*_*)*_*2*_*SO*_*4*_, ammonium sulfate; *NGQDs*, nitrogen-doped graphene quantum dots; *PBS*, phosphate-buffered saline; *PCMB*, 4-chloromercuribenzoic acid; *PdO*, palladium oxide; *RNA*, ribonucleic acid; *rGO*, reduced graphene oxide; *S*, sulfur; *SnS*_*2*_, tin(IV) sulfide; *SnS*_*2*_*@Ti*_*3*_*C*_*2*_, heteroconjuction of tin (IV) sulfide and titanium carbide MXene; *WS*_*2*_, tungsten disulfide; *Xe*, xenon; *ZnONRs/TNs/TiO*, heteroconjuction of zinc oxide nanorods and titanium dioxide; *λ*_*exc*_, excitation wavelength

Metallic nanostructures are highly valued in PEC systems for their surface plasmon resonance properties, which enhance light-particle interactions and improve photoelectric conversion efficiency [119]. However, their high cost and potential toxicity are significant drawbacks. In contrast, metal oxides are known for their strong light absorption, adjustable energy band-gap, and exceptional chemical stability, making them suitable for harsh environments and effective at increasing photocurrent signals, although they may suffer from charge carrier recombination losses [120]. On the other hand, carbon nitrides (g-C_3_N_4_) offer high chemical stability and ease of functionalization due to their two-dimensional (2D) structure and carbon–nitrogen conjugated bonds, but their relatively low conductivity can be a limitation [121]. Conversely, semiconductor quantum dots are appreciated for their quantum confinement effects, which enable size-tunable optoelectronic properties and efficient charge transfer [122]. However, they can encounter stability and toxicity issues. Finally, TMCs exhibit diverse optoelectronic properties and can function as metals and semiconductors, depending on their structure and conditions. They offer significant potential but face challenges with defect control and complex material synthesis [123].

Each type of nanomaterial has unique advantages and drawbacks, influencing its suitability for specific PEC applications. The properties of each photoactive nanomaterials play a crucial role, individually or as composite nanomaterials, in the assembly of biosensor platforms. These platforms leverage the unique virtues of each nanomaterial to enhance the detection device’s analytical properties. The selection of the spectral range of radiation in the PEC process depends on the wavelength at which each photoactive material in the platform absorbs the radiation and uses it in the PEC detection process. The following section briefly reports the mechanisms explored for each family of materials.

### Metals and metal oxides

The use of metallic nanostructures, such as those based on Au, Ag, and platinum (Pt), has been prompted in PEC systems due to their surface plasmon resonance properties [124]. Plasmons entail the collective oscillations of electrons on the surface of metallic nanoparticles. Electrons are excited when light interacts with these nanoparticles, generating plasmonic oscillations that produce a distinctive light-particle interaction [125]. This interaction leads to surface plasmon resonance, wherein light gets absorbed and scattered at particular wavelengths [126]. Plasmonic metal nanostructures can interact with light at frequencies aligned with the coherent oscillation of conduction electrons on the nanostructure’s surface, thus generating resonant surface plasmons [127–129]. Excitation with wide energy ranges favors the injection of hot electrons into the conduction bands of semiconductor materials through metal resonant plasmon energy transfer. The versatile optoelectronic attributes of plasmonic nanoparticles (narrow band-gap) enable photoelectric conversion efficiency through intimate interaction with wide band-gap semiconductors [130, 131].

Metal oxides constitute a class of nanomaterials with semiconducting characteristics ideal for applications in PEC devices. Metal oxides present strong light absorption, modulable charge carriers (electrons and holes), and extensive surface area available for electrocatalytic reactions [132, 133]. Metal oxides have broad and tunable energy band-gap, which allow them to absorb radiation in a wide range of wavelengths, a fundamental characteristic for generating electrons and holes upon material illumination [134]. Likewise, metal oxides have exceptional chemical stability and are suitable for operating in corrosive or hostile environments, such as PEC cells [135, 136]. Many metal-oxide nanomaterials have catalytic properties, accelerating electrochemical reactions and increasing photocurrent signals [137].

**Fig. 2:**
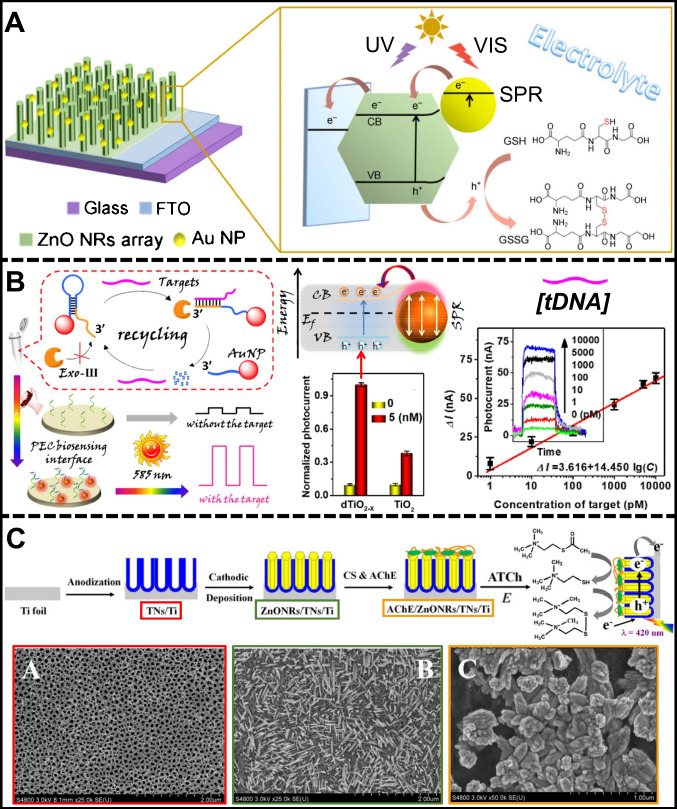
**A** Au/ZnO hybrid interface for PEC detection of GSSG, reproduced with permission from Ref. [99]. **B** PEC genosensor system based on dTiO_2−*x*_-AuNPs interaction for tDNA detection, reproduced with permission from Ref. [101]. **C** Fabrication of a PEC enzymatic sensor for elucidating the activity of AChE, reproduced with permission from Ref. [100]

Zhang et al. [99] conducted a glutathione detection assay utilizing a “photo-anode” founded on zinc oxide (ZnO) nanorods decorated with Au nanoparticles. This plasmonic nanoparticle/semiconductor hybrid was employed as a comparative and competitive test to elucidate the role of metallic nanoparticles as charge transducers induced by the injection of hot electrons into the ZnO conduction band. Investigating the pathways of PEC signaling was based on water oxidation, the reaction’s self-sustaining capability, and the detection of various glutathione concentrations. Figure 2* A* illustrates the detection mechanism of the Au/ZnO hybrid interface, where the surface plasmon resonance (SPR) of the Au nanoparticles enhances the absorption of visible plasmon-induced irradiation, generating energetic hot electrons. These electrons are then transferred to the conduction band of the metallic oxide material, facilitating charge transfer at the working electrode and enhancing charge carrier separation. Leveraging the surface sensitization provided by Au nanoparticles enables the creation of a glutathione disulfide (GSSG) detection assay with a linear range of 20–1000 µM, *R*^2^ = 0.996, and a LOD of 3.29 µM across the entire spectral window, encompassing both visible and ultraviolet ranges.

Conversely, the utilization of titanium oxide (TiO_2_) [10, 138] and its anatase phase (β-TiO_2_) [101] (Fig. 2B) has been explored for detecting specially designed DNA sequences within photoelectrode arrays. The plasmonic effect of an AuNP/tDNA nanobioconjugate on dTiO_2−*x*_ was employed for PEC detection of DNA. Likewise, exonuclease III (Exo III)-assisted target recycling amplification was coupled to the detection system to amplify the number of rDNA segments labeled with AuNPs. The capture probe targeted DNA sequences related to the manganese superoxide dismutase gene (MnSOD gene), a regulator of cellular redox homeostasis. AuNP-tagged hairpin DNA probes were designed to recognize target DNA (tDNA) and undergo hybridization, activating Exo III and leading to the digestion of the probes into residual DNA (rDNA) segments containing AuNPs. These segments were then anchored to the electrode surface, facilitating DNA analysis. When plasmonic nanoparticles and TiO_2_ converged within approximately 10 nm or less, a direct influence on the lifespan of charge carriers was observed. The generated hot electrons with a higher negative potential than that of the CB of dTiO_2−*x*_ could be injected smoothly into the CB, resulting in the enhancement of photocurrent. Moreover, the impact of the crystalline phase of TiO_2_ was demonstrated with a LOD of 0.6 pM, a linear range between 1 pM and 10 nM, and a high linearity (*R*^2^ = 0.967). This effect was rooted in the interplay between the nanomaterial structure of PEC processes and surface plasmons’ resonance, together with the injection of hot electrons into the semiconductor’s conduction band [139].

Zhang et al. [100] utilized a label-free PEC biosensing method to study acetylcholinesterase (AChE) activity using a nanocomposite made of zinc oxide nanorods (ZnONRs) within titanium dioxide nanotubes (TNs) on titanium foils (Fig. 2C). The PEC nanocomposite was created by anodic oxidation of Ti foil to form TNs, followed by cathodic deposition of ZnONRs. AChE immobilized on this nanocomposite showed enhanced photoelectrochemical responses under visible light. They observed that high concentrations of Cd^2+^ inhibited AChE activity, while low levels stimulated it. The PEC assay produced electron holes under light irradiation, which reacted with acetylthiocholine (ATCh) to generate thiocholine (TCh). It increased the photocurrent proportionally to the TCh concentration, reflecting AChE activity. The assay demonstrated high linearity in the 0.05–1000 µM range with a LOD of 0.023 µM. This method aided in understanding how metal ions affect enzyme activity and the pathogenesis of neurodegenerative disorders.

### Carbon nitrides

**Fig. 3:**
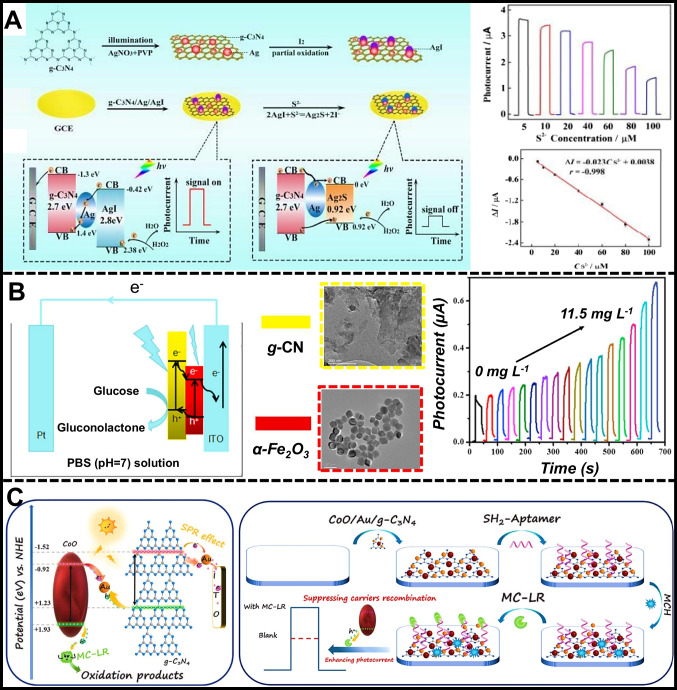
**A** GCE/g-C_3_N_4_/Ag/AgI assembly for the PEC detection of S^2−^ mean the Ag_2_S formation, reproduced with permission from Ref. [142]. **B** g-C_3_N_4_/α-Fe_2_O_3_/ITO heterojunction for the PEC detection of glucose, reproduced with permission from Ref. [144]. **C** PEC aptasensor assembly based on CoO/Au/g-C_3_N_4_ heterojunction for the MC-LR detection, reproduced with permission from Ref. [105]

Carbon nitrides are 2D nanostructures, often called g-C_3_N_4_, bearing a graphitic-like framework constituted by carbon and nitrogen atoms intricately assembled within a singular crystal lattice [140]. Their layered, planar configuration facilitates the establishment of carbon–nitrogen conjugated bonds, fostering the generation of a continuous network of delocalized electrons traversing the 2D structure and conferring semiconductor attributes [141]. This distinctive feature was harnessed by Zeng et al. [142], who devised a photoelectrode based on graphitic carbon nitride, silver, and silver iodide (g-C_3_N_4_/Ag/AgI) heterojunction, as illustrated in Fig. 3A. The integration of 2D g-C_3_N_4_ nanostructure with Ag as a plasmonic metal facilitated the design of a highly selective detection assay for hydrogen sulfide (H_2_S). The interaction of band-gap values, ranging between 2.7 eV (g-C_3_N_4_) and 2.8 eV (Ag/AgI), along with the strategic alignment of AgNPs, catalyzed electron transfer across metal/metal iodide and carbon nitride domains. The distribution of the three components on the platform formed a Z-scheme type system that reduced the recombination of photogenerated electron–hole pairs. The gradually increasing photocurrent showed that the Z-scheme pathway efficiently promoted the photoelectric conversion efficiency of g-C_3_N_4_. In the presence of target S^2−^, the AgI was transformed to Ag_2_S, leading to the broken Z-scheme electron migration pathway and, thus, the decreased photocurrent. The authors established that a 402-nm monochromatic radiation source was optimal for inducing the generation of hot electrons in plasmonic metals, their subsequent transfer to the 2D structure, and the acceleration of delocalized electrons. The optimal Z-scheme junction led to a highly effective PEC detection assay, exhibiting linearity between 5 and100 µM (*R*^2^ = 0.998) and a LOD of 1.67 µM. This phenomenon stemmed from the judicious selection of the excitation range, a facet substantiated by spectroscopic analyses performed on the photoelectrode [143].

The research of Xu et al. [144] also exploited the characteristics of an interface of g-C_3_N_4_ and α-Fe_2_O_3_ (Fig. 3B). This strategic pairing engendered a heterojunction for rapid migration of photogenerated carriers, thereby increasing the overall efficiency. The electrons within α-Fe_2_O_3_ could be effectively roused toward the conduction band, leveraging the influence of a 390-nm monochromatic radiation source to incite a gap formation within the valence band. Subsequently, the energized electron underwent a process of resonance energy transfer to the nanostructure of g-C_3_N_4_. The delocalized electrons gained momentum within this domain, participating in redox reactions in the medium. This assay highlighted the electron acceptor attributes inherent to graphitic carbon nitride structures, demonstrating high linearity (*R*^2^ = 0.993) in the range of 0.1–11.5 mg/L and a LOD of 0.03 mg/L. Combining g-C_3_N_4_ with other semiconducting or metallic materials produced exceptional photoactive nanocomposites ideal for supporting PEC detection assays [145].

Tan et al. [105] developed an aptamer-based PEC sensor (aptasensor) and a heterojunction composed of cobalt oxide (CoO), AuNPs, and g-C_3_N_4_ to detect microcystin-leucine arginine (MC-LR). The PEC platform, shown in Fig. 3C, enhanced the separation of photo-induced electron–hole pairs, and AuNPs significantly increased the visible light absorption through SPR. The heterojunction structure benefited from the large surface area of g-C_3_N_4_ and the tailored band-gap between g-C_3_N_4_ and CoO. AuNPs at the CoO-g-C_3_N_4_ interface enhanced light absorption and acted as electron mediators, forming a Z-scheme-type system that reduced charge carrier recombination. When MC-LR was captured on the PEC aptasensor, holes accumulated on the CoO VB, oxidizing MC-LR and further hindering electron–hole recombination, resulting in increased photocurrent. Visible light irradiation generated electrons on the CoO CB that flow to AuNPs, recombining with holes from the g-C_3_N_4_ VB, enhancing electron–hole pair separation and suppressing recombination. The SPR effect of AuNPs also produced hot electrons, contributing to increased photocurrent for MC-LR quantification, with a linear range of 0.1 pM to 10 nM, an *R*^2^ = 0.997, and a low LOD of 0.01 pM.

### Quantum dots (QDs)

Semiconductor QDs constitute a collection of nanoscale materials, typically encompassing 10^2^–10^5^ atoms, with dimensions not exceeding 10 nm [146, 147]. Their compactness engenders an environment conducive to the quantum confinement of electrons and holes across all three spatial dimensions [148]. Consequently, QDs harbor a distinctive semiconductor property wherein the energies and wave functions of the constrained quantum states can be manipulated by adjusting the QDs’ size, shape, and composition. This inherent confinement is pivotal for exceptionally efficient charge transfer [149, 150].

**Fig. 4:**
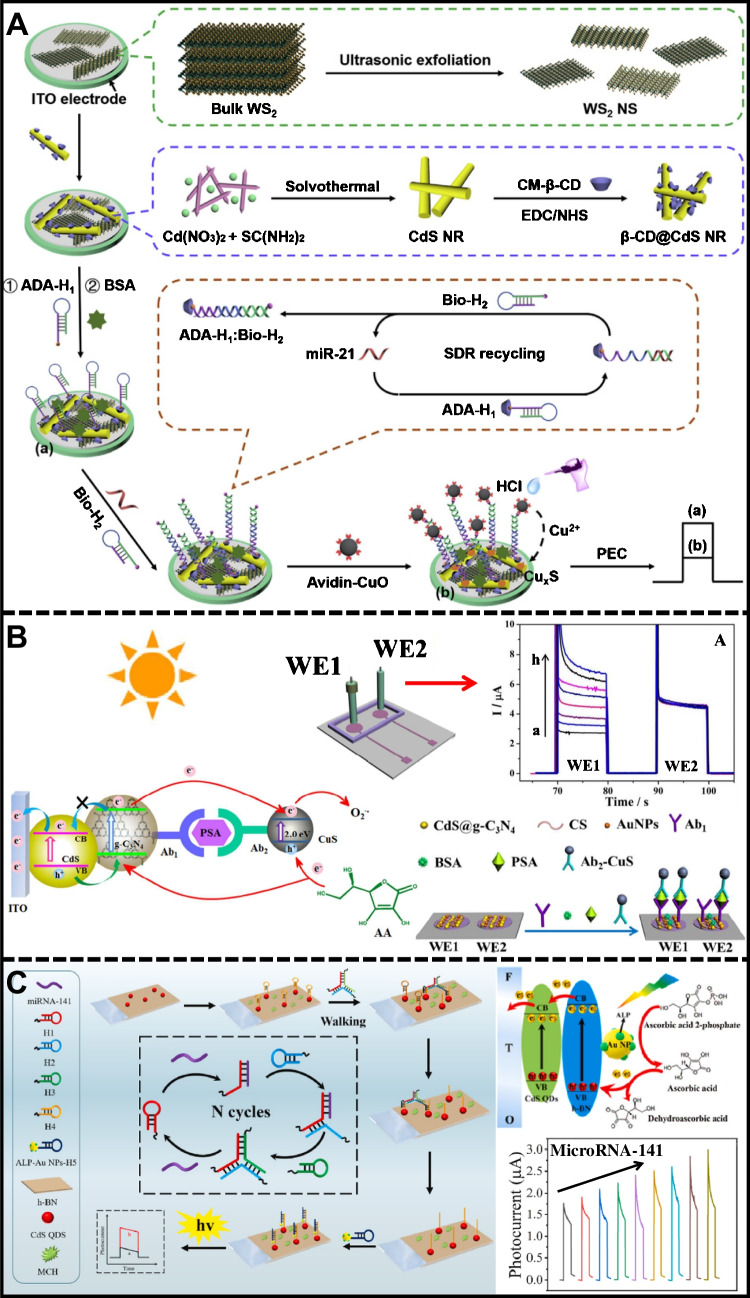
**A** WS_2_/β-CD@CdS assembly for the PEC detection of miR-21, reproduced with permission from Ref. [113]. **B** PEC immunosensor based on ITO/CdS/g-C_3_N_4_/CuS heterojunction for the PSA detection, reproduced with permission from Ref. [109]. **C** FTO/CdS/h-BN/AuNPs heterojunction platform for the PEC detection of miRNA-141, reproduced with permission from Ref. [112]

Xue et al. [113] demonstrated the PEC behavior of QDs using a photoelectrode composed of tungsten disulfide (WS_2_), β-cyclodextrin (β-CD), and cadmium sulfide (CdS) heterostructure (Fig. 4A). Incorporating CdS QDs increased the photocurrent due to their ability to generate holes and electrons, which were enhanced by quantum confinement effects and created a localized electric field for ascorbic acid (AA) oxidation. The experiment used a variable power radiation source covering the visible spectrum and specific ultraviolet frequencies, highlighting the narrow wavelength activation range of QDs. The nanostructured interface was utilized to construct an ultrasensitive PEC biosensor for detecting microRNA-21 (miR-21) using a cyclic strand displacement reaction (SDR)-mediated Cu^2+^ quenching mechanism. Adamantane (ADA)-labeled hairpin DNA1 (ADA-H1) was immobilized on the electrode via host–guest interaction with β-CD@CdS. When a mixture of target miR-21 and biotin-labeled hairpin DNA2 (Bio-H2) was added, ADA-H1 unfolded through hybridization. Bio-H2 then hybridized with ADA-H1, releasing miR-21 and triggering another SDR process. Avidin-labeled CuO nanoparticles attached to the duplex were dissolved, releasing Cu^2+^, which reacted with CdS to form Cu_x_S, reducing the photocurrent. This easy-to-assemble WS_2_/β-CD@CdS heterojunction and the SDR-dependent Cu^2+^ quenching signal cascade enabled highly sensitive miR-21 detection, with a highly linear range of 0.1 fM to 10 pM (*R*^2^ = 0.997) and a LOD of 25.1 aM.

In a similar vein, Liu et al. [109] developed a label-based PEC biosensing method for detecting prostate-specific antigen (PSA) using a CdS@g-C_3_N_4_ heterojunction and CuS-conjugated antibodies (Ab_2_-CuS) for signal amplification (Fig. 4B). The PEC immunosensor was constructed by assembling CdS@g-C_3_N_4_, chitosan (CS), AuNPs, and primary antibodies (Ab_1_) on dual electrodes, followed by blocking unbound sites with bovine serum albumin (BSA). Varying concentrations of PSA were added to one working electrode (WE1) and a fixed concentration to the other (WE2) before incubating Ab_2_-CuS on both. The specific binding of PSA to Ab_2_-CuS led to a weakened photocurrent response in a linear concentration range of 0.01–50 ng/mL and a LOD of 4 pg/mL. Spatial resolved radiometry was based on the photocurrent intensity ratio between WE1 and WE2. With well-matched band energies, the photoactivity of the CdS core and g-C_3_N_4_ shell enabled effective light harvesting and electron–hole pair separation. Electrons migrated to the CdS CB while holes transferred to the g-C_3_N_4_ VB, enhancing photoactivity and stability. The Ab_2_-CuS conjugates acted as signal amplifiers by weakening the PEC intensity in the presence of PSA. This effect occurred due to photogenerated electrons transferring from g-C_3_N_4_ to CuS, reducing electron transfer to the electrode. The captured electrons formed O_2_^−•^ with dissolved O_2_, enabling ultrasensitive PSA detection through photocurrent generation.

QDs coupled to highly sensitive and label-free PEC biosensors were also studied by Yu et al. [112], as shown in Fig. 4C. The PEC biosensor was based on CdS QDs sensitized porous hexagonal boron nitride (h-BN) nanosheets (NSs) and multiple-site tripodal DNA walkers (TDWs) formed through catalytic hairpin assembly (CHA). The porous h-BN NSs provided a large surface area and numerous active sites, making them ideal for photoelectric substrate materials. The h-BN/CdS QDs composite ensured the efficient transmission of photogenerated electrons and holes, resulting in high photoelectric conversion efficiency. CHA-formed TDWs triggered by miRNA-141 immobilized a significant amount of alkaline phosphatase (ALP) on the electrode surface, catalyzing ascorbic acid 2-phosphate (AAP) to produce AA as an electron donor. The h-BN/CdS QDs composite was coupled to a fluorine-doped tin oxide (FTO) electrode and modified with Hairpin4 (H4) DNA tracks. Upon miRNA-141 initiation, TDWs bound to H4 on the electrode surface and underwent strand displacement, exposing the toe region of H4. This region formed a double-stranded DNA structure with ALP-AuNPs-H5 through further strand displacement, continuing the walking process and anchoring more ALP on the electrode. Under visible light, h-BN NSs and CdS QDs photogenerated electrons and holes, moving electrons from the CB of h-BN to CdS QDs and then to the electrode, creating a stable photocurrent. It allowed for the sensitive detection of miRNA-141, achieving an excellent linear range from 1 fM to 100 nM (*R*^2^ = 0.997) and a detection limit of 0.73 fM. This PEC biosensor provides a robust strategy for early clinical diagnosis and biomedical research.

### Transition metal chalcogenides (TMCs)

TMC nanomaterials are composed of chalcogen atoms, commonly oxygen, sulfur, selenium, or tellurium, in conjunction with a transition metal [151, 152]. Extensive research has been conducted to explore the optoelectronic properties of TMCs, especially tungsten disulfide (WS_2_) and MoS_2_. The molecular arrangement of TMCs involves positioning metal atoms surrounded by chalcogen atoms in an organized manner, forming 2D or 3D layers [153]. Due to the specific arrangement of atoms within the structure, they can exhibit conductive characteristics under certain conditions, such as nanometer-scale thinning or introducing defects [154, 155].

**Fig. 5:**
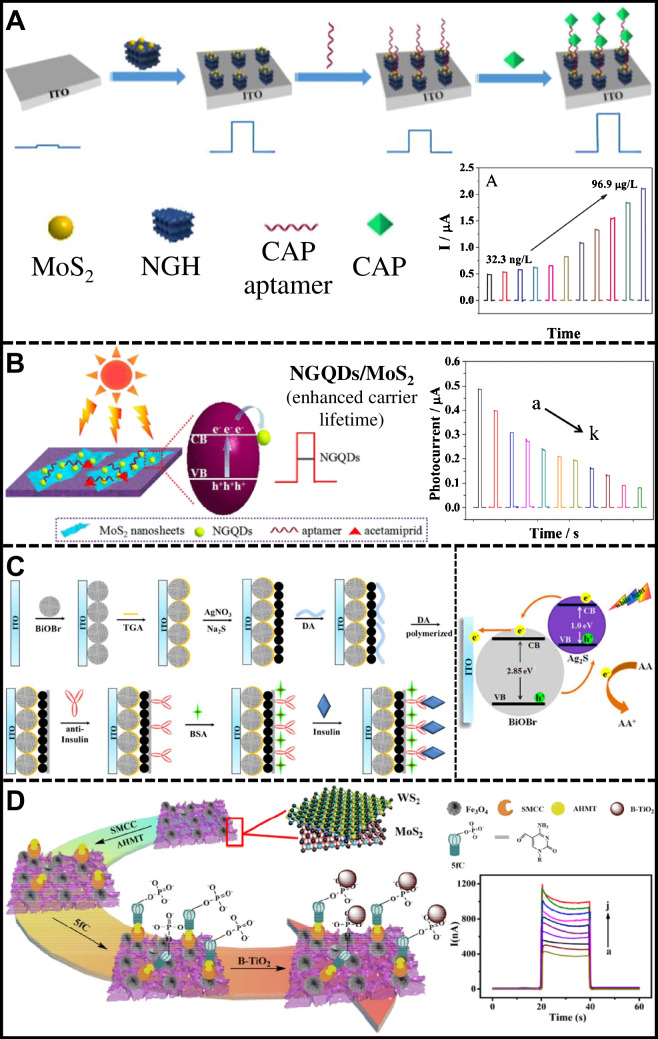
**A** PEC aptasensor based on ITO/NGH/MoS_2_ for CAP detection, reproduced with permission from Ref. [156]. **B** MoS_2_/NGQDs-modified platform for PEC aptasensing detection of acetamiprid, reproduced with permission from Ref. [110]. **C** PEC immnosensing assembly for insulin detection based on ITO/BiOBr/Ag_2_S heterojunction, reproduced with permission from Ref. [157]. **D** WS_2_/MoS_2_/Fe_3_O_4_/β-TiO_2_ platform for PEC detection of 5fC, reproduced with permission from Ref. [117]

Wang et al. [156] and Dai et al. [116] reported improved performance of TMCs through the synergy of MoS_2_/N-graphene (Fig. 5A) and MoS_2_/NGQDs (Fig. 5B) nanostructures, respectively. Both studies utilized semiconductors to sensitize the TMCs and capture signals within narrow wavelength ranges of approximately 400 and 630 nm. Wang et al. employed MoS_2_/N-graphene (NGH) heterojunctions for PEC analysis of chloramphenicol (CAP) in food samples with the aid of a CAP aptamer. The MoS_2_/NGH composites displayed a reversed “V-shaped” p-n heterojunction curve, promoting efficient spatial charge separation and longer photocarrier lifetimes. The PEC sensor recognized CAP quickly, inhibiting electron–hole recombination and enhancing the photocurrent. The sensor showed excellent linearity from 32.3 ng/L to 96.9 μg/L (*R*^2^ = 0.998), with a detection limit of 3.23 ng/L. On the other hand, Dai et al. used nitrogen-doped graphene quantum dots (NGQDs) with ultrathin MoS_2_ nanosheets (NGQDs/MoS_2_) to create a high-performance photoactive material. The NGQDs extended the lifetimes of photogenerated charge carriers, leading to improved charge separation and substantial photocurrent signal amplification for acetamiprid detection. The photocurrent intensity decreased with increasing acetamiprid concentration, showing a linear range from 0.05 pM to 1 nM and a detection limit of 16.7 fM. These advancements highlight the benefits of TMCs in PEC detection, including chemical stability, efficient charge carrier separation, and transport, resulting in significantly improved detection performance.

One particular type of TMC is metal sulfides, a class of nanomaterials that manifest metallic and semiconductor properties ideal for electronic applications [158, 159]. The optoelectronic mechanism of these materials hinges on the role of metal cations as electron donors and sulfide anions as electron acceptors [160], resulting in a partially occupied valence band and an unoccupied conduction band. This dynamic engenders a distinctive band-gap contingent upon the structural attributes of the ionic arrangement within the crystal lattice [11, 161]. One clear example is given by Wei et al. [157]. They developed a highly sensitive insulin detection assay on bismuth oxybromide (BiOBr) and silver sulfide (Ag_2_S)-modified indium tin oxide (ITO) electrodes (Fig. 5C). The photoelectrode was irradiated with 420-nm monochromatic light in a solution with AA as a redox probe and PBS as an electrolyte medium. The resonant energy levels of the BiOBr microspheres and Ag_2_S nanoparticles enabled efficient electronic transition under visible light with high photocurrent signals compared to the individual systems. The photocurrent response in the PEC system decreased with a progressive increase in the insulin concentration on the electrode in a range between 0.001 to 20 ng/ml, *R*^2^ = 0.993, and a detection limit of 0.2 pg/ml. This method ensured measurement stability and robust PEC activity.

Likewise, ITO photoelectrodes were modified with a heterojunction of nanosheets of tungsten disulfide, molybdenum disulfide, and titanium dioxide (WS_2_/MoS_2_/β-TiO_2_) to detect 5-formylcytosine (5fC), as shown in Fig. 5D [117]. The nanostructured surface of TMCs was coated with Fe_3_O_4_-NH_2_ covalently coupled to 4-amino-3-hydrazino-5-mercapto-1,2,4-triazole (AHMT) using a cross-linker of N-succinimidyl 4-(N-maleimidomethyl) cyclohexanecarboxylate (SMCC). The hydrazine of AHMT specifically captured 5fC by reaction with the aldehyde groups of the AHMT/Fe_3_O_4_/WS_2_/MoS_2_/ITO interface. AA was used as a redox probe for interference-free detection under white light. 2D metal sulfide-semiconductor heterojunctions demonstrated outstanding photoactive and analytical performance, with a linear range of 0.01–200 nM (*R*^2^ = 0.998) and a LOD of 2.7 pM. It highlights the role of TMCs in PEC sensing applications, providing sensitive and time-stable responses.

In the evolving field of PEC bioanalysis, significant progress has been made across various approaches and applications, each offering unique advantages and challenges. Zhao et al. [42] emphasized integrating PEC techniques with biomolecular detection, highlighting the development of bismuth-based photoelectrodes to address toxicity and low efficiency in conventional materials. This approach shows promise in enhancing PEC performance through improved charge separation and light absorption. On the other hand, Ai et al. [43] focused on applying electrochemical, electrochemiluminescent, and PEC techniques for detecting epigenetic modifications, underscoring the importance of these methods in diagnosing diseases and understanding biological functions. It emphasized the need for ultra-sensitive and specific detection technologies in this context. Similarly, Chen et al. [44, 45] provided an extensive overview of PEC DNA biosensors, detailing the types of transducers and probe immobilization techniques used and various DNA interactions that can be monitored. Despite significant advancements, challenges such as stability and reproducibility remain, with future research directed to solve such issues, develop new photoactive materials, and integrate nanotechnology for clinical applications.

Liu et al. [162] explored the advancements in self-powered PEC sensors, which enhance portability and simplify operation by eliminating the need for external power sources. These sensors leverage solar energy to drive redox reactions, offering superior sensing performance and environmental benefits. In contrast, Pang et al. [163] delved into semiconductor nanomaterial-based PEC biosensing, highlighting the role of materials such as metallic oxides, sulfides, and graphitic carbon nitride in constructing high-performance PEC sensors. It pointed out the challenges of improving photoconversion efficiency and addressing photobleaching. Finally, Tang et al. [79] emphasized the impact of nanotechnology on PEC biosensing, focusing on advanced photoactive nanomaterials and their charge separation and transfer mechanisms, the biomedical applications of PEC biosensors, and the potential of composite materials in overcoming limitations like high charge recombination rates and low photoelectric conversion efficiency. Overall, the promising future of PEC bioanalysis, driven by continuous innovations in material science and sensing mechanisms, aims to enhance sensitivity, specificity, and practical applications in fields ranging from disease diagnosis to environmental monitoring.

## Red light and NIR excited PEC biosensors

The evolution of diverse structural configurations integrating optical and electrochemical analyses sets the stage for the refinement of more accurate and efficient PEC assays to quantify a wide array of substances [164]. Within this framework, the adoption of red light and NIR excitation in PEC devices offsets the limitations of existing sensors with UV light. Radiation in the UV range restricts the applications of PEC biosensors in areas of biodetection of clinically relevant biomarkers due to conformational damage and decreased biological activity of protein-type bioreceptors such as antibodies or enzymes [165–167]. NIR light, spanning wavelengths from over 650 up to 1700 nm, is gaining importance in biosensing and biomedicine due to its minimal spectral interference, ability to penetrate deep tissues, and limited harm to biological entities [168, 169]. Consequently, considerable efforts have been devoted to extending the excitation source into the visible spectrum by coupling small band-gap semiconductors to augment light absorption efficiency and biosensor performance. Radiation in this range is less energetic, facilitating non-invasive or minimally invasive detection in biological samples such as blood or tissues [170, 171]. Red light and NIR PEC biosensors also exhibit reduced background interference (photobleaching), which improves signal quality, biosensor sensitivity, and probe stability over extended analysis periods [172]. Table 2 reviews the most representative reports on nanobiosensors activated by red and NIR light.

**Table 2:** PEC biosensors activated by red light and NIR

Platform structure	Electrolyte/redox probe	λ_exc_ (nm)	Detected biomarker	Linear range	LOD	Ref
ITO/WS_2_/AuNPs	PBS/AA	630	MCF-7 cell	10^2^ –5 × 10^6^ cells/mL	21 cells/mL	[173]
ITO/AgS_2_/AuNPs	PBS/AA	810	MCF-7 cell	10^2^ – 10^7^ cells/mL	100 cells/mL	[174]
FTO/NaYF_4_:Yb,Tm@TiO_2_	G bases	980	CEA	0.01–40 pg/mL	3.6 pg/mL	[175]
GC/AuNSs	PBS	780	AA	0.1 – 11 mM	10 µM	[176]
ITO/Bi_2_O_2_S/AuNPs	PBS/AA	808	MCF-7 cell	50 – 5 × 10^6^ cells/mL	17 cells/mL	[177]
FTO/NaYF_4_:Yb,Tm/ZnO/CdS	PBS/AA	980	AFP	0.01–200 ng/mL	5 pg/mL	[178]
FTO/CdS/NaYF_4_:Yb,Tm@NaYF_4_	PBS	980	miRNA-21	0.05–100 nM	8 pM	[179]
FTO/Ag_2_S/AuNP	PB	980	MC-LR	10 pg/L –10 μg/L	7 pg/L	[180]
ITO/AgInS_2_	Tris–HCl/AA-NaCl-KCl	630	CCRF-CEM cell	1.5 × 10^2^–3 × 10^5^ cells/mL	16 cells/mL	[56]
NaYF_4_:Yb,Tm@ZnO	Na_2_SO_4_	980	CEA	0.1–300 ng/mL	0.032 ng/mL	[181]
NaYF_4_:Yb,Er/Ag_2_S	Na_2_SO_4_	980	CEA	0.005–5 ng/mL	1.9 pg/mL	[165]
TiO_2_/AuNPs	PBS	760	TET	2–150 nM	0.6 nM	[182]
ITO/CN/TsCuPc	PB/DA	> 630	DA	0.05–50 µM	2 nM	[183]
FTO/ZnO/Ag/NaYF_4_:Yb,Tm	PBS	980	AFP	0.05–100 ng/mL	0.04 ng/mL	[184]
NaYF_4_:Yb,Er@CdTe	Na_2_SO_4_	980	CEA	10 pg/mL – 5.0 ng mL	4.8 pg/mL	[185]
FTO/NaYF_4_:Yb, Er@Au@CdS	Na_2_SO_4_/glucose-H_2_O_2_	980	AFP	0.01–40 ng/mL	5.3 pg/mL	[186]

*AFP*, alpha-fetoprotein; *AuNSs*, gold nanostars; *AA*, ascorbic acid; *AgInS*_*2*_, silver indium disulfide quantum dot; *AgS*_*2*_*/AuNPs*, heteroconjuction of silver sulfide quantum dot and gold nanoparticles; *Bi*_*2*_*O*_*2*_*S/AuNPs*, heteroconjuction of bismuth oxysulfide chalcogenide and gold nanoparticles; *CEA*, carcinoembryonic antigen; *CN/TsCuPc*, heteroconjuction of carbon nitride and copper phthalocyanine; *DA*. dopamine; *FTO*, fluorine-doped tin oxide; *G*, guanine; *GC*, glassy carbon; *H*_*2*_*O*_*2*_, hydrogen peroxide, *ITO*, indium tin oxide; *KCl*, potassium chloride; *λ*_*exc*_, excitation wavelength; *miRNA-21*, microRNA 21; *Na*_*2*_*SO*_*4*_, sodium sulfate; *NaCl*, sodium chloride; *NaYF*_*4*_, *Er@CdTe*, core–shell sodium yttrium tetrafluoride doped with ytterbium and erbium, coated with cadmium telluride upconversion nanoparticle; *NaYF*_*4*_, *Tm@TiO*_*2*_, core–shell sodium yttrium tetrafluoride doped with ytterbium and thulium, coated with titanium dioxide upconversion nanoparticle; *NaYF*_*4*_, *Tm/ZnO/CdS*, heteroconjuction of sodium yttrium tetrafluoride doped with ytterbium and thulium upconversion nanoparticle, zinc oxide, and cadmium sulfide; *PB*, phosphate-buffered; *PBS*, phosphate-buffered saline; *TET*, *Tris–HCl*, Tris(hydroxymethyl)aminomethane hydrochloride; *WS*_*2*_*/AuNPs*, heteroconjuction of tungsten disulfide and gold nanoparticles

Red and NIR light have been explored to detect breast cancer cell lines (MCF-7) [173, 174]. Plasmonic nanoparticles were incorporated into ITO electrodes modified with multicomponent semiconductor nanomaterials to improve the photoelectric conversion efficiency. In the first study, TMC, WS_2_, and AuNPs heterojunctions were assembled on ITO to detect MCF-7 cells non-invasively. A long excitation wavelength was employed in PEC bioanalysis to prevent cell damage or denaturation. WS_2_ nanosheets exhibited low cytotoxicity and harvested red light to produce photoinduced electrons injected into the ITO electrode, with photogenerated holes and scavenged by AA. The AuNPs assembly on WS_2_ nanosheets amplified the photocurrent by approximately 31 times due to the localized surface plasmon resonance (LSPR) effect of the AuNPs. The direct transfer of hot electrons from the plasmonic metal to the CB of the WS_2_ nanosheet occurred by the induction of a collective oscillation of free electrons on the surface of the AuNPs under 630-nm irradiation (Table 2). A MUC1 aptamer immobilized to the nanostructured interface was used to capture MCF-7 cells as a model analyte specifically. Detection of MCF-7 cells was related to the decrease in photocurrent under irradiation with red light at a fixed voltage in amperometry at 0.1 V, showing a high linearity in a range of 10^2^ – 5 × 10^6^ cells/mL (*R*^2^ = 0.996), with a LOD 21 cells/mL. The efficiency of plasmon-enhanced photoelectric conversion highlighted the effectiveness of PEC methods for sensitively detecting cancer-related biomarkers without collateral damage to the analyte biomolecules.

**Fig. 6:**
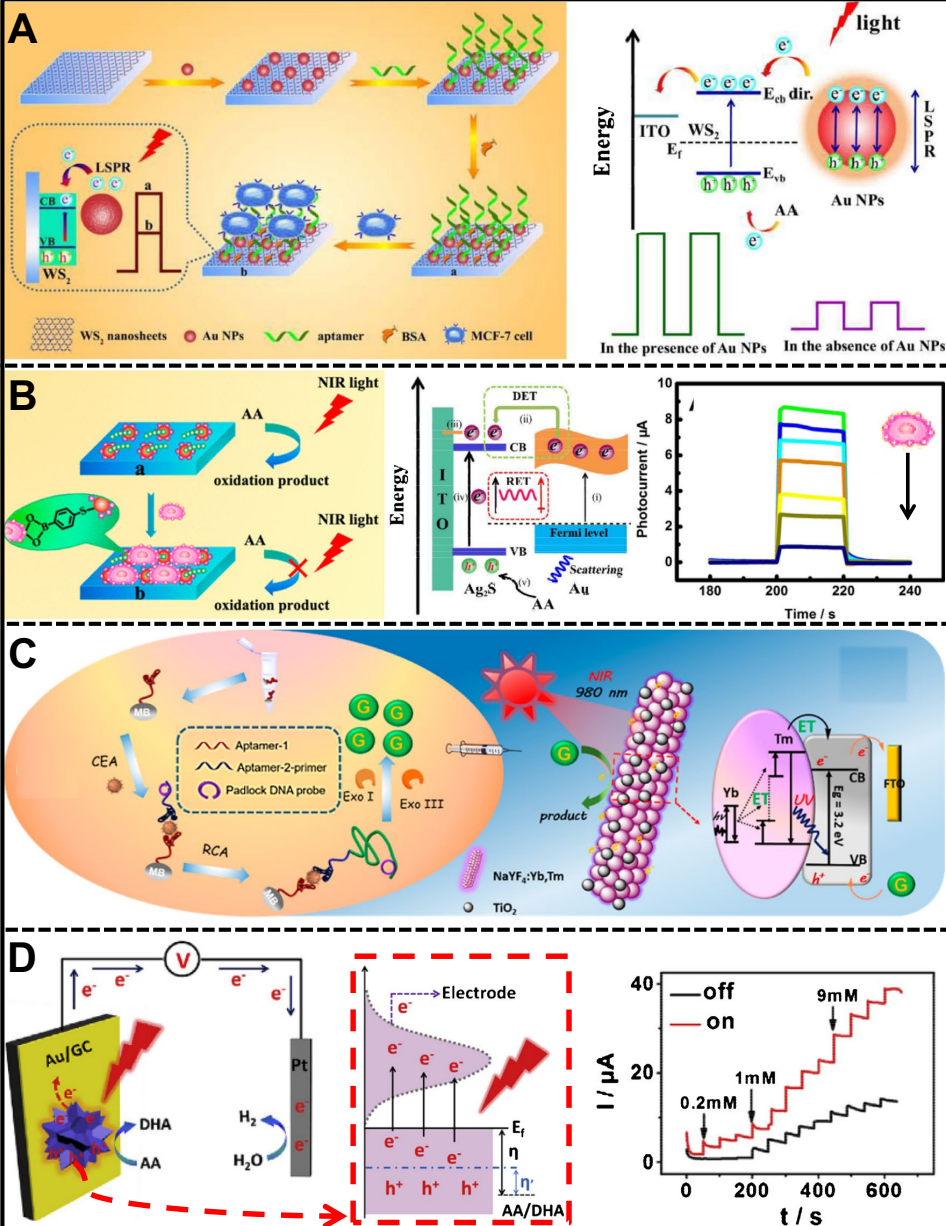
**A** WS_2_/AuNPs-modified platform for PEC cytosensing detection of MCF-7, reproduced with permission from Ref. [173]. **B** PEC cytosensing detection of MCF-7 based on ITO/Ag_2_S/Au heterojunction, reproduced with permission from Ref. [174]. **C** FTO/NaYF_4_:Yb,Tm@TiO_2_ platform for PEC detection of CEA, reproduced with permission from Ref. [175]. **D** PEC detection of variable concentrations of AA based on GC/AuNS heterojunction, reproduced with permission from Ref. [176]

On the other hand, the ITO/Ag_2_S/AuNPs heterojunction was used under 810-nm NIR light to quantify MCF-7 cells and dynamically evaluate cell surface glycan expression after sialidase (SA) stimulation, as shown Fig. 6B and Table 2. Ag_2_S QDs showed excellent PEC properties in the NIR range, and adding AuNPs created a hybrid material with enhanced photoelectric conversion efficiency. AuNPs exhibited strong LSPR, leading to significant signal amplification. The biosensing platform featured a self-assembled monolayer (SAM) of thiol on the AuNPs, facilitating the assembly of 4-mercaptophenylboronic acid (MPBA) molecules. MPBA was a biorecognition element to capture MCF-7 cells through the reaction between SA on the cell membrane and boric acid in MPBA. This specific capture decreased photocurrent proportional to the MCF-7 concentration, with a linear range of 10^2^ – 10^7^ cells/mL, an *R*^2^ = 0.992, and a 10^2^ cells/mL LOD. The LSPR effect enhanced the photoelectric conversion efficiency by increasing light scattering and promoting electron–hole pair generation in Ag_2_S QDs. The platform effectively transferred plasmonic energy from AuNPs to Ag_2_S QDs, improving light absorption and charge separation, which is crucial for sensitive MCF-7 detection.

The plasmon-enhanced direct electrocatalysis of gold nanostars (AuNSs) deposited on a glassy carbon (GC) substrate for PEC detection of AA is shown in Fig. 6D [176]. The electrocatalytic performance of the AuNSs/GC system increased substantially under red light irradiation. This enhancement was attributed to the collective oscillations of conduction electrons in the light-excited AuNSs, also called LSPR. The study highlights the tunability of the LSPR of plasmonic nanostructures through parameters such as size, shape, interparticle distance, and surrounding medium properties. LSPR excitation drove electrons from the sharp tips (hot spots) of the AuNSs to higher energy levels, generating hot electrons. The anisotropic AuNSs hosted numerous “hot spots,” facilitating the efficient generation of hot carriers and a reduced activation energy barrier. Likewise, the photothermal effect of LSPR excitation further increased the electrocatalytic performance of the AuNSs. The measurement at open circuit potential (OCP) led the hot electrons to the external circuit, separating them from the holes and preventing recombination. The accumulation of hot holes on the surface of AuNSs enhanced the oxidation ability toward AA, reducing the overpotential and activation energy for AA electrocatalysis in a linear range of 0.1 – 11 mM with a LOD of 10 µM and a detection sensitivity of 190.9 µA/cm^2^mM. The detailed description of plasmon-mediated electrocatalysis under NIR and red-light irradiation lays the foundation for the design of PEC (bio)sensors based on anisotropic plasmonic nanostructures.

Similarly, other studies have reported the use of the conjunction between AuNPs and TMC, QDs, carbon nitrides, or metallic oxides, activated with red or NIR radiation for the detection of various targets shown in Table 2: MCF-7 cells at 808 nm [177], MC-LR cells at 980 nm [180], CCRF-CEM cells at 630 nm [56], tetracycline at 760 nm [182], and dopamine at 630 nm [183]. These studies highlight the versatility of detection modalities achievable with different arrangements of photoactive nanomaterials using red and NIR radiation. Additionally, future research can focus on developing new NIR light-sensitive materials and miniaturized photoelectrodes, applying them further for in vivo and single-cell analysis due to the versatility of irradiating nanostructured surfaces based on these photoactive nanomaterial arrangements.

Lanthanide-doped up-conversion nanoparticles (UCNPs) represent another material-sensitive NIR radiation type. UCNPs convert low-energy excitation light into high-energy fluorescence emission, leveraging their exceptional chemical stability, resistance to photobleaching, low toxicity, and ability to convert NIR light into shortwave light in the UV–visible spectral range. UCNPs typically consist of a host material like NaYF_4_ doped with lanthanide ions such as Er^3+^, Yb^3+^, and Tm^3+^, which possess discrete energy levels. Upon NIR illumination, these lanthanide ions absorb low-energy photons through sequential multi-photon absorption or energy transfer processes. A common mechanism, energy transfer upconversion (ETU), involves an ion like Yb^3+^ absorbing a photon and transferring its energy to another ion like Er^3+^, allowing the absorption of multiple low-energy photons. In a typical two-photon upconversion process, a lanthanide ion absorbs two photons sequentially, first exciting the ion from the ground to an intermediate state and then to a higher energy state. The absorbed energy is often transferred from a sensitizer ion (e.g., Yb^3+^) to an activator ion (e.g., Er^3+^), which emits a higher-energy photon. Once in the excited state, these ions can return to lower energy states by emitting photons (radiative relaxation), observed as upconversion luminescence, while minimizing non-radiative relaxation to maintain high upconversion efficiency. The application of PEC biosensors based on NIR radiation of UCNPs for detecting biomarkers in the clinical field has also been demonstrated. Tang et al. [175] presented a proof of concept of a PEC platform for the sensitive detection of carcinoembryonic antigen (CEA) under 980-nm NIR excitation, using core–shell NaYF_4_:Yb,Tm@TiO_2_ UCNPs. (Fig. 6C). The detection strategy was based on light conversion from NIR to UV and signal amplification by rolling circle amplification (RCA). The platform employed a sandwich assay with two CEA-targeting aptamers immobilized on biofunctional magnetic beads, activating RCA to produce a long guanine (G)-rich oligonucleotide strand. Enzymatic digestion released G bases by enhancing the photocurrent under NIR light excitation. This approach took advantage of the minimal photobleaching and low phototoxicity of NIR light by efficiently converting it to UV light to activate the TiO_2_ layer and generate a photocurrent increase proportional to the CEA concentration. The device exhibited high sensitivity with an LOD of 3.6 pg/mL, in a linear range of 0.01–40 pg/mL (*R*^2^ = 0.994), and successfully detected CEA in serum samples. This novel PEC biosensing system is promising for detecting low-abundance biomolecules in biological fluids using UCNPs.

UCNP-activated systems have been extensively used for PEC biosensing due to their ability to function as non-invasive sensitizer systems activated by 980-nm radiation, which in turn activates heterojunction systems between UCNPs and metals, metal oxides, and QDs through visible radiation emitted via fluorescence processes. The detection of alpha-fetoprotein (AFP) has been achieved through the heterojunction between NaYF_4_:Yb,Tm/ZnO/CdS [178] and NaYF_4_:Yb,Er@Au@CdS [186], as shown Table 2. Additionally, the detection of carcinoembryonic antigen (CEA) has been conducted using UCNP heterojunctions based on NaYF_4_:Yb,Tm@ZnO [181], NaYF_4_:Yb,Er/Ag_2_S [165], and NaYF_4_:Yb,Er@CdTe [185]. These studies demonstrate the versatility of such systems for analyte detection based on the conjunction of different types of materials in hybrid systems, which enhance the detection performance of PEC systems and pave the way for ongoing research into nanostructured platforms based on UCNPs.

## Characterization of PEC biosensors

The accurate and rigorous characterization of PEC interfaces is crucial in developing reproducible and trustworthy detection assays. The most widely used techniques for characterizing PEC biosensing are listed in Table 3. Typically, the most relevant parameters of PEC platform surfaces are characterized in terms of surface chemistry, morphology, and (photo)electrochemical performance. Energy-dispersive X-ray spectroscopy (EDS) is a powerful analytical technique for characterizing PEC biosensors. It provides valuable information about the elemental composition, material characterization, surface modification verification, quality control, material degradation studies, and correlation with materials within the PEC system [96]. Furthermore, Fourier-transformed infrared spectroscopy (FT-IR or Raman) and X-ray photoelectron spectroscopy (XPS) are versatile analytical techniques that can be integrated into PEC biosensors to provide insights into molecular composition, chemical bonds, surface functionalization, and the monitoring of chemical changes. FT-IR and XPS enhance the understanding of PEC biosensor behavior by offering information about the chemical nature of the sensor’s surface and the biomolecule-analyte interactions [187, 188].

**Table 3:** Characterization techniques of PEC biosensing interfaces

Properties	Characterization technique	Use in PEC systems	Ref
*Surface chemistry*	EDX	Backscattered electrons in electron microscopy are employed to obtain elemental mapping of the composition of the PEC interface	[96]
	FTIR-Raman	The functional groups available for anchoring photoactive nanomaterials and biological recognition elements are characterized by measuring the different vibrational modes determined by the bonds of atoms from these groups	[187]
	XPS	XPS offers the ability to characterize the PEC interface’s chemical composition accurately. It is also helpful in monitoring the biosensor assembly based on the types of bonds formed	[188]
	UV–vis DRS	This technique leads to the characterization of solid interfaces by dispersing a fraction of the incident UV–vis radiation on its surface, as seen in PEC systems with photoactive nanomaterials	[189]
	PL	The photoactivity of materials nanostructured on the PEC biosensing interface is characterized by photoluminescence (PL), which involves the spontaneous emission of light from a material under optical excitation	[190]
*Morphology*	SEM-FESEM	The modification of PEC interfaces with nanostructured photoactive materials can be characterized using SEM by scanning with secondary and backscattered electrons. Furthermore, FESEM with field emission can be used to attain higher resolution, improving the observation of nanoscale details	[191]
	AFM	Critical morphological properties, such as surface topography, interaction forces, mechanical properties, electrical properties, and biomolecular interactions, can be measured at PEC sensing interfaces	[117]
*Electrochemistry*	CV	CV can be used to investigate redox reactions in the PEC biosensor and to measure the photocurrent generated when light activates the photoactive material in the presence of the analyte. This technique measures the photocurrent response across a range of potentials, enabling the determination of redox potentials and reaction kinetics	[192]
	EIS	EIS is employed to analyze the electrical impedance of the PEC system over a range of frequencies. It can provide insights into charge transfer resistance, adsorption processes, and other electrochemical properties relevant to PEC biosensing	[193]
	Chronoamperometry	This technique involves measuring the photocurrent at a fixed potential over a specific period. By monitoring changes in photocurrent over time, chronoamperometry can provide kinetic information about the interaction between the analyte and the bioactive elements on the sensor surface	[194]

Alternatively, ultraviolet–visible diffuse reflectance spectroscopy (UV–vis DRS) is a valuable analytical technique employed in PEC biosensors to investigate the optical properties of materials, specifically their absorption and reflectance of ultraviolet and visible light. This technique is essential for band-gap determination, quantification of photogenerated carriers, monitoring chemical changes, studying the kinetics of PEC reactions, and characterizing the performance of functionalized surfaces [189]. Finally, photoluminescence (PL) can be utilized in PEC biosensors to investigate the emission of light, usually fluorescence, from materials exposed to photons, typically from a light source. PL is commonly used for characterizing fluorescent labels, enhancing sensitivity, monitoring redox reactions, conducting kinetic studies, enabling multiplexed detection, and facilitating real-time monitoring of PEC surfaces [190].

Scanning electron microscopy (SEM), field emission scanning electron microscopy (FESEM) [191], and atomic force microscopy (AFM) [117] are powerful surface analytical techniques that can be used in PEC biosensors to study the surface morphology, structure, and composition of materials. Together, they provide comprehensive analyses of morphology, nanostructuring, chemical composition, real-time monitoring, and interaction analysis during the immobilization of biomolecules. Electrochemical techniques, including cyclic voltammetry (CV), electrochemical impedance spectroscopy (EIS), and chronoamperometry, play crucial roles in developing and characterizing PEC biosensors. CV is relevant for determining redox properties, measuring band-gaps and energy levels, kinetic studies, and assessing sensitivity in PEC devices [192]. On the other hand, EIS is used to characterize interfacial properties, monitor charge transfer resistance, and understand charge transfer rates and diffusion processes [193]. Finally, chronoamperometry is commonly used for real-time monitoring and steady-state current measurements [194].

The surface chemistry, morphology, and structural properties of nanostructured materials that alter the interfaces in PEC biosensors are meticulously characterized to optimize the analytical performance of these devices. Transmission electron microscopy (TEM) is a powerful technique used to investigate nanoscale structures and compositions, offering exceptional resolution and the ability to observe internal structures [195]. X-ray diffraction (XRD) is a fundamental tool in materials research and crystallography, providing detailed information about atomic arrangements in crystals, which is essential for understanding material properties at the atomic scale [196]. Dynamic light scattering (DLS) and electrophoretic light scattering (ELS) are typically employed to study size and surface charge [197] for characterizing colloidal systems.

## Concluding remarks and perspectives

PEC analysis and ongoing research in photoactive materials as transduction platforms have garnered extensive attention to enhance these devices’ analytical performance. It is achieved by addressing the inherent challenges of PEC detection systems, focusing on acquiring new nanomaterials, and designing novel detection strategies. For example, nanomaterials capable of facilitating energy interconversion processes with superior efficiency have boosted the ultrasensitive, reproducible, and stable detection of various bio-analytes. Optoelectronic properties of nanomaterials exhibiting semiconductor behavior, including various metal oxides, carbon nitrides, QDs, and TMCs, have been extensively exploited for this purpose. However, challenges still must be tackled fully by a broader range of excitation sources covering more portions of the visible and NIR spectral range. Therefore, detection strategies aimed at enhancing the PEC behavior of devices have shifted toward sensitizing the materials with counterparts excitable at longer wavelengths and lower energy levels. Adopting red light and NIR excitation in PEC devices may overcome the limitations of existing (bio)sensors primarily reliant on UV–vis light that restricts their potential applications, particularly in vivo, due to its shallow tissue penetration. NIR light, spanning wavelengths greater than 650 nm, enjoys minimal spectral interference, deep tissue penetration, and limited damage to biological entities.

The possibility of miniaturizing detection assays is another strength of PEC devices, enhancing electrode design versatility without compromising performance metrics like electron transport and stability. Miniaturization enables multi-analyte detection in single measurements, which is essential for POC devices that improve disease diagnosis and intervention. Leveraging patient-specific biology, physiology, and genetic precision medicine promises to revolutionize healthcare by predicting disease risks and treatment responses. In this context, transformative diagnostics incorporate smart, innovative devices and informatic approaches using big data analytics, the Internet of Things (IoT), machine learning, blockchain, artificial intelligence (AI), augmented reality, system integration, cloud and fog computing, and smartphones, offering advanced healthcare solutions through cutting-edge converging technologies. Integrating PEC devices with these advanced systems enhances their capability to deliver precise and rapid multi-analyte detection in real-time, which is crucial for effectively implementing precision medicine. However, most research involving PEC devices for biosensing assays employs spectral ranges in the tail of the UV region, the near-UV–visible region, or a combination of the entire visible region, overlooking the significant advantages of red light and the NIR range. Integrating these underutilized spectral ranges could further enhance the sensitivity and effectiveness of PEC devices in advanced smart diagnostic applications.

The advantages of using metal oxides and carbon nitrides in photoelectrochemical biosensors are substantial. As described by conventional band theory, metal oxides offer wide-space ionic structures with minimal curvatures in their electronic bands, resulting in smaller effective masses and enhanced carrier mobility. Conversely, carbon nitrides provide an adjustable band-gap for tunable electrical conductivity, light response, and high transparency across a broad spectrum of wavelengths, making them ideal for PEC detection devices. These properties make metal oxides and carbon nitrides valuable in advancing PEC biosensors, enhancing their performance, and expanding their applications in various fields. Their high carrier charge mobility holds promise for high-speed electronic devices like thin film transistors and photovoltaic devices. Furthermore, their photoluminescent properties facilitate light emission upon electromagnetic radiation excitation, proving useful in (bio)sensors and lighting devices. Their high mechanical strength makes them ideal for optical and electronic devices requiring robust and durable materials.

Semiconductor QDs activated by UV radiation offer optoelectronic properties such as tunable size, high photoluminescence quantum yield, quantum confinement effect, and strong absorption coefficients. Their high excitation efficiency enables effective absorption and conversion of UV light into visible emission, making them ideal for light-emitting devices. The adjustable emission spectrum of QDs, achieved by varying their size, is valuable for biosensors, displays, and as marks of biomolecules. Their stability and durability ensure consistent performance over time under various conditions, and their compatibility with flexible substrates allows for use in flexible electronic and optoelectronic devices like wearable displays and (bio)sensors. TMCs with high excitation efficiency also convert UV light into visible light, which is helpful for light emission devices. Their wide adjustable band-gap range enhances versatility, and their stability and good light dispersion improve the uniformity and quality of emitted light in lighting and display applications.

Exploiting the benefits of PEC systems activated at wavelengths exceeding 650 nm is worth mentioning. Their profound tissue penetration, cellular safety, and detector stability capabilities enable the detection of biomolecules within dense samples, including tissues and bodily fluids, rendering them promising for biomedical and diagnostic applications. Additionally, they effectively mitigate autofluorescence, thereby increasing sensitivity and selectivity by minimizing interference from biological components. Moreover, these systems inflict minimal damage to cells and tissues, facilitating real-time measurements under physiological conditions without adverse effects. For example, notable optoelectronic properties of plasmonic nanoparticles enhance light capture and conversion efficiency through plasmonic coupling, thereby increasing detection sensitivity and enabling the detection of biomolecules at low concentrations. Energy UCNPs can convert infrared light into visible or ultraviolet light, allowing biosensors to be excited with shorter wavelengths and enhancing detection efficiency by reducing the autofluorescence of biological components. NIR-activated QD, which absorbs NIR light and emits visible light, is helpful for exciting PEC biosensors, thereby improving the sensitivity and selectivity of biomolecule detection. Photonic crystals manipulate and control light propagation at specific wavelengths, improving light capture efficiency and detection sensitivity.

Furthermore, PEC systems feature photodetectors characterized by enhanced stability, ensuring precise and reproducible measurements over extended periods. Their integration with biosensing approaches, such as optical coherence tomography and in vivo fluorescence imaging, paves the way for further positioning these devices into imaging systems tailored for biomedical applications. These technologies promise to develop more sensitive, selective, and efficient PEC biosensors for biomedical, food safety, and environmental applications, thus revolutionizing clinical diagnostics, pathogen detection, and environmental monitoring, ultimately improving society’s health and well-being.

## Data Availability

No datasets were generated or analyzed during the current study. Data will be made available on request.
